# A Novel WRKY Transcription Factor, *MuWRKY3* (*Macrotyloma uniflorum* Lam. Verdc.) Enhances Drought Stress Tolerance in Transgenic Groundnut (*Arachis hypogaea* L.) Plants

**DOI:** 10.3389/fpls.2018.00346

**Published:** 2018-03-16

**Authors:** Kurnool Kiranmai, Gunupuru Lokanadha Rao, Merum Pandurangaiah, Ambekar Nareshkumar, Vennapusa Amaranatha Reddy, Uppala Lokesh, Boya Venkatesh, A. M. Anthony Johnson, Chinta Sudhakar

**Affiliations:** ^1^Plant Molecular Biology Unit, Department of Botany, Sri Krishnadevaraya University, Anantapur, India; ^2^Department of Plant, Food, and Environmental Sciences, Dalhousie University, Truro, NS, Canada

**Keywords:** *MuWRKY3* TF, drought stress tolerance, transgenic groundnut, stress-responsive genes, ROS, antioxidative metabolism

## Abstract

Drought stress has adverse effects on growth, water relations, photosynthesis and yield of groundnut. WRKY transcription factors (TFs) are the plant-specific TFs which regulate several down-stream stress-responsive genes and play an essential role in plant biotic and abiotic stress responses. We found that *WRKY3* gene is highly up-regulated under drought stress conditions and therefore isolated a new *WRKY3TF* gene from a drought-adapted horsegram (*Macrotyloma uniflorum* Lam. Verdc.). Conserved domain studies revealed that protein encoded by this gene contains highly conserved regions of two WRKY domains and two C2H2 zinc-finger motifs. The fusion protein localization studies of transient *MuWRKY*3-YFP revealed its nuclear localization. Overexpression of *MuWRKY3* TF gene in groundnut (*Arachis hypogaea* L.) showed increased tolerance to drought stress compared to wild-type (WT) plants. *MuWRKY3* groundnut transgenics displayed lesser and delayed wilting symptoms than WT plants after 10-days of drought stress imposition. The transgenic groundnut plants expressing *MuWRKY3* showed less accumulation of malondialdehyde, hydrogen peroxide (H_2_O_2_), and superoxide anion (O_2_^∙-^), accompanied by more free proline, total soluble sugar content, and activities of antioxidant enzymes than WT plants under drought stress. Moreover, a series of stress-related *LEA, HSP, MIPS, APX, SOD*, and *CAT* genes found up-regulated in the transgenic groundnut plants. The study demonstrates that nuclear-localized *MuWRKY3* TF regulates the expression of stress-responsive genes and the activity of ROS scavenging enzymes which results in improved drought tolerance in groundnut. We conclude that *MuWRKY3* may serve as a new putative candidate gene for the improvement of stress resistance in plants.

## Introduction

The abiotic stresses such as drought, heat, salt, and cold are the major causes for declined crop productivity worldwide. Combat to the stress, plants have evolved a sophisticated physiological and molecular networks. Drought is a severe environmental factor that significantly restricts the plant growth and productivity (Surendran et al., 2017). Drought stress causes changes in the cell membrane, osmolytes accumulation, inhibit photosynthesis traits, decrease biomass production and yield components in crop plants. (Binott et al., 2017). At the molecular level, several TFs like AP2/EREBP, NAC, WRKY, bZIP, MYB, and bHLH play a vital role in regulating downstream genes to protect plants from drought stress (Hennig, 2012). The WRKY TFs are one of the predominant families of plant-specific regulatory proteins in the plant kingdom and are known to participate in biotic and abiotic stress responses (Rushton et al., 2010; Liu et al., 2015; Sarris et al., 2015; Joshi et al., 2016). TFs play a vital role in stress responses by interacting with specific *cis-*acting elements present in gene promoters thereby regulating expression of down-stream elements (Birkenbihl et al., 2017; Chen et al., 2017). All WRKY TFs contain one or two conserved domains having approximately 60 amino acid residues with a conserved heptapeptide WRKYGQK, and a C2H2 or C2HC zinc finger-like motif (Eulgem et al., 2000). WRKY domain binds to the TTGACC/T W-box of the target gene and regulates its transcription (Brand et al., 2013; Bakshi and Oelmüller, 2014). WRKY TFs have been classified into three groups based on the number of WRKY domains present and the features of their zinc finger motif (Rushton et al., 2010; Rinerson et al., 2015a). Each domain of group I WRKY TFs appear to be functionally diverse; the C-terminal domain helps in specific binding to the W box, and the N-terminal domain is involved in increasing the affinity (Eulgem et al., 2000). In normal growth conditions, some members of WRKY TFs play regulatory roles like trichome development (Bakshi and Oelmüller, 2014), root growth (Grunewald et al., 2013), seed development (Schluttenhofer and Yuan, 2014), leaf senescence (Miao and Zentgraf, 2007; Rinerson et al., 2015b), dormancy (Ding et al., 2014), and secondary metabolites biosynthesis (Eulgem et al., 2000). In general, WRKY TFs can play multiple roles in plants by regulating the downstream related genes either positively or negatively (Dai et al., 2015).

The majority of deduced WRKY TFs are well-known for their functions in biotic stress response. Expression of *NtWRKY3* and *NtWRKY6* TFs was reported in *Nicotiana attenuata* during herbivore attacks (Skibbe et al., 2008). Lai et al. (2008) reported the elevated transcript abundance of *AtWRKY3* and *AtWRKY4*, in *Arabidopsis* infected with *Botrytis cinerea*. Currently, research focus is on the functional characterization of WRKY proteins in response to abiotic stresses. Either up-regulation or down-regulation of an individual WRKY gene can result in improved abiotic stress tolerance (Wang et al., 2013; Dai et al., 2015). Several WRKY TF gene family members responsive to abiotic stresses have also been reported WRKY TF genes like *WRKY25, WRKY26*, and *WRKY33* were recognized to regulate cross-talk between ethylene and heat shock protein response related signaling pathways (Li et al., 2011). A multiple abiotic stress responsive *TaWRKY10* up-regulated during PEG, NaCl, cold, and H_2_O_2_ treatment was reported in wheat (Wang et al., 2013). In contrast to other abiotic stresses, few WRKY TFs were identified specifically to drought stress response. The expression of WRKYs in response to drought such as *AtWRKY57* from *Arabidopsis* (Jiang et al., 2012), *GsWRKY20* from soybean (Luo et al., 2013), and *ZmWRKY58* from maize (Cai et al., 2014) have been investigated. The overexpression of WRKY genes in plants showed enhanced tolerance to several abiotic stresses. For example, overexpression of *OsWRKY11* in rice displayed an improved tolerance to high temperature and salt (Wu et al., 2009). Also, overexpression of several cotton WRKY genes (*GhWRKY17, GhWRKY34*, and *GhWRKY41*) showed an increased salt and drought tolerance in tobacco (Yan et al., 2014; Chu et al., 2015; Zhou et al., 2015). Further, overexpression of two wheat WRKY genes (*TaWRKY19* and *TaWRKY93*) in *Arabidopsis* confers higher tolerance to salt and drought stress (Niu et al., 2012; Qin et al., 2015). These genes conferred tolerance to abiotic stresses in plants through detoxification of cytotoxic compounds (ROS and RCC scavenging), with improved osmotic adjustment, maintaining membrane stability, and by regulating the stress-responsive genes. Though, a few researchers reported the role of WRKY TFs in abiotic stress responses in model plants, the functions of most WRKY TFs from non-model plants remains underexplored. Horsegram, a drought-adapted grain-legume has the unique ability to grow in poor soils under receding moisture conditions. Groundnut is important oil yielding seed-legume crop grown in semi-arid regions of the world in rain-fed areas and is frequently encounters to drought spells of different duration and intensities. Considering the importance of groundnut in dry-land agriculture, its tolerance to drought raises an important issue for groundnut improvement programs. Keeping in mind the various functions of WRKY TFs under complex environmental conditions and to further understand the function of WRKY TFs, we isolated and funcationally characterized a drought stress-responsive *MuWRKY3* gene from horsegram. Transgenic groundnut plants overexpressing *MuWRKY3* exhibited improved drought stress tolerance compared to wild-type (WT) suggesting that *MuWRKY3* can serve as a new candidate gene for improving the tolerance to drought stress in crop plants.

## Materials and Methods

### Plant Material and Stress Treatments

The pot-cultured horsegram plants were grown for 19-days in botanical garden under natural photoperiod (10–12 h; 27 ± 4°C). Stress treatments included were, (1) drought stress imposed to plants by withholding water and leaf samples were collected at wilting stage, (2) salt stress imposed by adding 2% NaCl solution to the pots and collected samples at 72 h after treatment, (3) for dehydration stress, detached leaves were allowed to dry on filter paper in ambient conditions for 8 h, (4) for cold stress treatment potted plants were subjected to 10°C for 48 h, and (5) for heat stress treatment plants were subjected to 50°C for 8 h in a temperature controlled growth chamber. The samples were flash frozen in liquid nitrogen and stored at -80°C for further analysis.

### RNA Extraction and Quantitative Real-Time PCR Analysis

Total RNA from horsegram leaf samples was extracted using TRIzol reagent method. DNase treatment of extracted total RNA was performed using the TURBO DNA-free kit (Ambion, United States). 200 ng of RNA was used to synthesize cDNA and subsequently used as a template for PCR and quantitative RT-PCR analysis (Hu et al., 2012). Expression analysis was performed using Real-time PCR-System (AB StepOne, United States) with three biological replicates and actin as an internal control. The relative quantification was studied using 2^-ΔΔCt^ method (Livak and Schmittgen, 2001) to evaluate quantitative variations in transcript level between three replicates. Primers used in the RT-PCR assay are included in (Supplementary Table S1).

### *MuWRKY3* Gene Cloning and Phylogenetic Analysis

Gene-specific primers were used to amplify the full-length gene from horsegram cDNA (Supplementary Table S1). The amplified full-length product was cloned into pTZ57R/T vector and sequenced. WRKY3 sequences from different plants were downloaded from Plant TF Database and compared with the *MuWRKY3* sequence. Multiple sequence alignment was done using ClustalX software. Unrooted Neighbor-joining Tree used to perform the phylogenetic tree analysis. Tree view software produced a graphical representation, and internal branching support was estimated using 1000 bootstrap replicates and 111 random odd numbers. Conserved domains were predicted by using NCBI conserved domain database. Molecular weight and isoelectric point were predicted using online software Expasy^1^. Subcellular localization was predicted by Plant-mPLoc 2.0^2^ (Chen et al., 2010) (Supplementary Figure S4).

### Vector Construction and Plant Transformation

A gene construct overexpressing *MuWRKY3* was generated by cloning full-length gene to binary vector pCAMBIA2301 under a constitutive 35S promoter. *Agrobacterium* EHA105 is carrying CaMV35S:*MuWRKY3* with kanamycin as a selectable marker was used for plant transformation. Transgenic plants were generated by *in planta* transformation (Rohini and Rao, 2001). *MuWRKY3* gene fused with YFP (35S:*MuWRKY3*::YFP) was used for localization studies. *MuWRKY3* gene was cloned in pDONOR207 (Invitrogen) using gateway cloning method, and the gene was subsequently cloned into a binary vector pAM-PAT-p35S-YFP (Bernoux et al., 2008). The plant expression vector expressing pAM-PAT-p35S-*MuWRKY3*-YFP and pAM-PAT-p35S-YFP were transformed into *Agrobacterium tumefaciens* strain EHA105.

### Sub Cellular Localization and Transactivation Assay

*Agrobacterium* EHA105 carrying pAM-PAT-p35S-*MuWRKY3*-YFP and pAM-PAT-p35S-YFP were introduced into *Nicotiana benthamiana* leaves using a needle-less syringe (Li et al., 2014). After 48 h of incubation, the leaf samples were observed for YFP signal. YFP excitation was performed at 515 nm, and emission was spotted in the range of 525–600 nm using a confocal laser scanning microscope (Olympus, FV1000, Germany). The transactivation activity of *MuWRKY3* genes was investigated by yeast one-hybrid assay using strain AH109. The vector pGBKT7 carrying *MuWRKY3* (pBD), His-tag reporter gene (pGBKT7-*MuWRKY3*) and pBD were transformed into yeast, following Clontech yeast protocol handbook. Colony PCR was performed for selecting the positive colonies. The yeast strains were streaked on SD/-Trp and SD/-His plates containing five mM 3-amino-1, 2, 4-triazole (3-AT) for transcriptional activity assay.

### Analysis of Gene Integration and Overexpression

The seeds harvested from T0 plants were sown on MS medium containing 200 mg/L kanamycin, and the kanamycin survived plants were selected as true transformants (Herrera-Estrella et al., 1983). The presence and integration of transgene were further confirmed by PCR analysis from genomic DNA using gene-specific WRKY3, NptII and GUS primers (Supplementary Table S1). GUS enzyme activity was assessed in seedlings of putative transformants and WT (Jefferson, 1987). After confirmation, positive transgenic plants were advanced to further generations. Putative transgenic plants were confirmed by PCR amplifications to select T2 and T3 seeds. QRT-PCR used to quantify transcript abundance of the transgene. Southern blot analysis was done to confirm the stable transgene integration in T3 generation transgenic line. Genomic DNA digested with, BamHI at 37°C and GUS probe was used for analysis. Blotting was performed using Biotin-labeled PCR amplified gene fragment of GUS probe (Dai et al., 2015). The hybridized membrane was washed and detected according to manufacturer’s instructions (Thermo Scientifics Biotin DecaLabel DNA Labeling Kit, Germany).

### Stress Tolerance Assays of Transgenic and WT Plants

To assess the drought tolerance, pot grew 30-day-old groundnut transgenic, and WT plants were subjected to drought stress by withholding water for 10 days. Phenotypic differences between WT and transgenic groundnut plants were recorded. Reactive oxygen species, superoxide anions were measured according to Doke (1983) and hydrogen peroxide content by Singh et al. (2006), lipid peroxidation were estimated by measuring the MDA content according to Hodges et al., 1999. Leaf free proline content was measured by Ninhydrin method (Bates et al., 1973). Leaf total soluble sugars were measured by Arnon (1949) and antioxidative enzyme activities, SOD by Giannopolitis and Ries (1977) and APX by Nakano and Asada (1981) were determined sectrophotometrically (Shimadzu UV-1800, Japan).

### Statistical Analysis

To check the significant differences in all the physiological and biochemical experiments were subjected to the SPSS statistic data software (version 16.0) and evaluated using one-way ANOVA *post hoc* multiple comparisons from the Duncan’s test at a significance level of *P* ≤ 0.05.

## Results

### Expression Patterns of MuWRKY3 Under Various Stress Treatments

**FIGURE 1:**
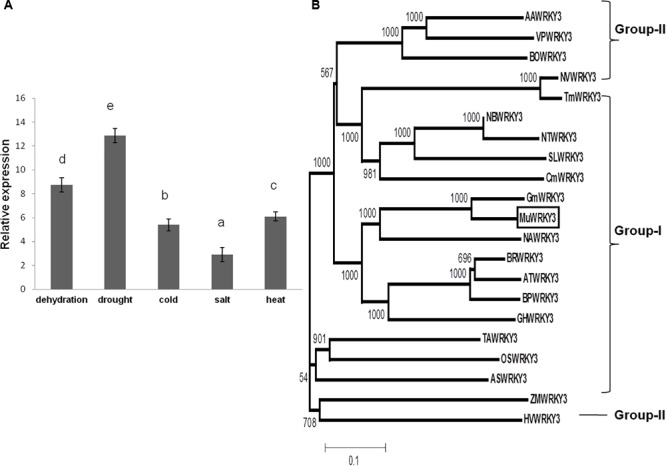
**(A)** Expression of *MuWRKY3* in horsegram under various abiotic stresses as determined by real-time PCR analysis. Error bars represent standard deviations (SD) for three independent replicates with different letters at *P* < 0.05 significant level. **(B)** Unrooted Bootstrapped Neighbor Joining tree analysis of *MuWRKY3* and WRKY protein sequences from different species. The amino acid sequences of the conserved WRKY domain region were aligned using ClustalX version 2.1. *MuWRKY3* is boxed in the figure (Supplementary Figure S5).

The quantitative RT-PCR analysis was performed to determine the transcript abundance patterns of *MuWRKY3* in leaf tissues of horsegram under unstressed and several imposed abiotic stresses. The results showed that expression of *MuWRKY3* was detected in leaf tissues under dehydration, drought, heat, salt, and cold treatments. Although the WRKY3 gene was highly up-regulated in all stress conditions, its expression was significantly higher under drought stress condition (**Figure 1A**) than under other stresses. Salt stress (200 mM NaCl) treatment caused an increase in *MuWRKY3* transcript abundance by 3.4-fold, heat stress by 6.1-fold, low temperature (cold) treatment led to up-regulation by 5.0-fold. During dehydration and drought treatments, the transcription of *MuWRKY3* performed a significant up-regulation by 9.4- and 13.5-fold, respectively.

### Cloning and Sequence Annotation Analysis of MuWRKY3

Horsegram (*Macrotyloma uniflorum* Lam. Verdc.) is well adapted to semi-arid conditions with higher drought tolerance ability. From, our previous study, the *MuWRKY3* gene was found to be highly up-regulated among eight different WRKY genes studied (Kiranmai et al., 2016). In this study, the full-length WRKY3 gene was successfully isolated from horsegram and designated as *MuWRKY3* (GenBank Accession: KM520390.1). Sequence analysis presented that the *MuWRKY3* gene is 1476 bp with coding a deduced protein of about 490 amino acids with molecular weight of 53.7 kDa and an isoelectric point of 6.12. *MuWRKY3* protein conserved domain analysis displayed that the presence of two WRKY domains and a C2-H2 zinc finger motif and belongs to group I of WRKY TF superfamily (Rushton et al., 2010; Huang et al., 2012) (Supplementary Figures S1–S3).

### Phylogenetic Relationship of MuWRKY3 With Other WRKY Proteins

To characterize the divergence of the isolated *MuWRKY3* protein with the other plant WRKY3 proteins, domain proteins were analyzed by ClustalX alignment (**Figure 1B**). The phylogenetic analysis revealed that *MuWRKY3* was clustered into group I of the WRKY TF family and most closely associated to *GmWRKY3* than from *NaWRKY3, GhWRKY3, AtWRKY3, BpWRKY3*, and *BrWRKY3*.

### MuWRKY3 Gene Localizes to Nucleus and Exhibit Transcriptional Activity

**FIGURE 2:**
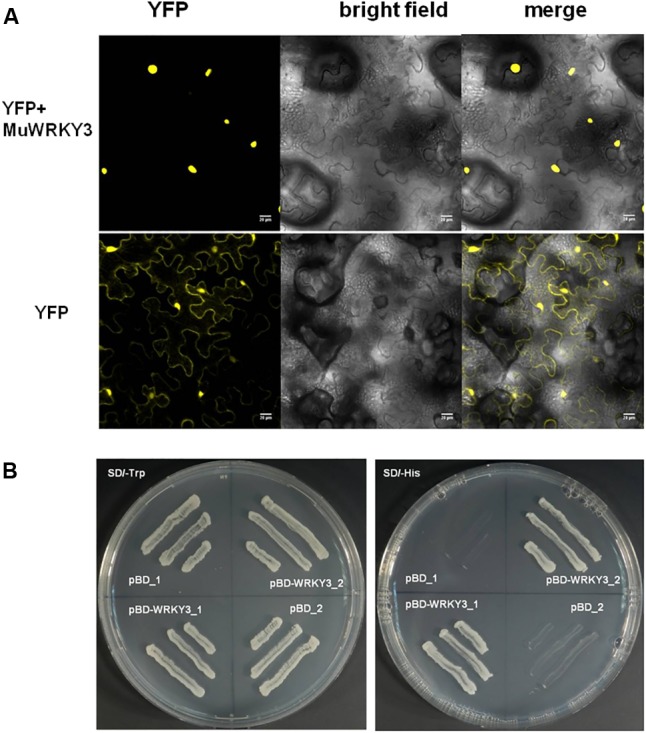
**(A)** Sub-cellular localization of YFP and *MuWRKY3:*YFP in the epidermal peels of *Nicotiana Benthamiana*. **(B)** Transactivation activity analysis of *MuWRKY3* was performed using yeast strain AH109. The transformants pBD*-WRKY3* and pBD (pGBKT7) were streaked on SD/-Trp and SD/-His plates containing 5 mM 3-amino-1, 2, 4-triazole (3-AT).

To determine the subcellular localization of the *MuWRKY3* gene was fused to N-terminal with YFP reporter gene that was expressed under the constitutive promoter (35S CaMV). The Agrobacterium harboring 35S::*MuWRKY3*-YFP and 35S::YFP constructs was infiltrated to 3-week-old tobacco leaves by needleless syringe. The microscopic image clearly shows that *MuWRKY3*-YFP protein is accumulated in the nucleus and the YFP alone occurred all over the cell organelles including cytoplasm and nucleus (**Figure 2A**). Further, yeast transactivation assay was carried out for *MuWYKY3* gene in yeast cells revealed the activation of His-reporter gene in the presence of *MuWRKY3* protein. The yeast cells transformed with pBD-*MuWRKY3* grew well in SD/-Trp and SD/-His plates containing 5 mM 3-amino-1, 2, 4-triazole (3-AT) where pBD could survive in SD/-Trp medium only (**Figure 2B**). Together these results suggest that *MuWRKY3* is nuclear-localized protein serving as a TF.

### Generation of Transgenic Groundnut Plants Overexpressing MuWRKY3

**FIGURE 3:**
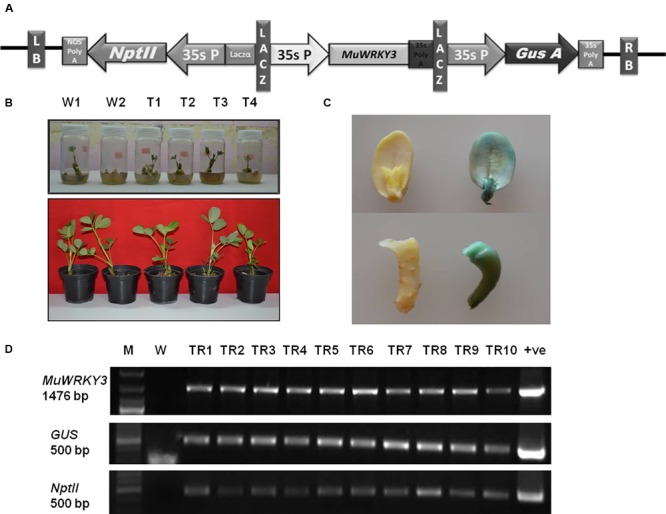
**(A)** A linear map of *MuWRKY3* overexpressing vector with GUS and NptII marker genes for screening, 35S promoter and polyA terminator. Entire expression cassette is flanked by left and right borders in pCAMBIA2301. **(B)** Screening of transgenic and WT plants on half strength MS medium containing 200 mg/lit kanamycin and their acclimatization. **(C)** GUS histochemical staining of WT and transgenic seedlings at T_3_ generation. **(D)** PCR Amplification of *MuWRKY3* gene *GUS* and *NptII* gene from genomic DNA of transgenic and WT plants: M, gene ruler mix; WT, wild-type; TR1 to TR10, transgenic lines.

To study the functional relevance of *MuWRKY3* gene in response to drought stress the groundnut transgenic expressing *MuWRKY3* driven by the CaMV 35S promoter was developed (**Figure 3A**). A tissue culture independent *Agrobacterium* mediated *in planta* transformation method was adopted to develope the groundnut transgenics (Rohini and Rao, 2001). The putative transformants were analyzed in T1, T2, and T3 generation for the resistance to kanamycin (**Figure 3B**), drought tolerance and integration of the gene by PCR. Lines resistant to kanamycin were selected and advanced next generation (Supplementary Figure S6). The transgenics were also confirmed for the activity of glucoronidase due to the expression of Gus gene. The transgenic cotyledons clearly demonstrate that the higher activity of glucoronidase (**Figure 3C**). The presence of the transgene in transgenic groundnut plants in T3 generation confirmed by PCR analysis using gene-specific primers of WRKY3, GUS, and NptII(**Figure 3D**).

### Transgenic Groundnut Plants Expressing MuWRKY3 Showed Improved Drought Tolerance

**FIGURE 4:**
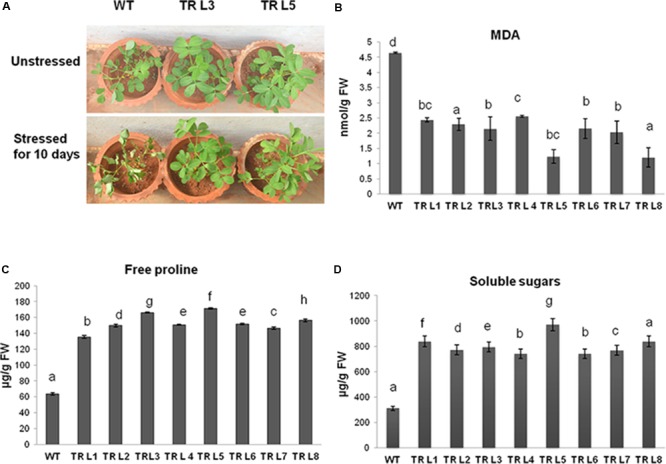
**(A)** Phenotypic differences in transgenic lines and WT groundnut plants under unstressed and drought stressed for 10 days. **(B)** MDA content in WT and transgenic plants under stress. **(C)** Free proline content and **(D)** total soluble sugars in transgenic and WT plants under drought stress conditions (WT- WT, TRL1 to TRL8 – Transgenic lines). Data is analyzed using SPSS version 16.0, values shown is the mean of three replicates and ±*SE* of three replicates and letters shown above the bars are significantly different at *P* < 0.05 (DMR).

Under unstressed conditions, all groundnut lines showed no visible phenotypic differences (unpublished data). However, WT and independent T3 lines exposed to drought stress for 10 days showed stress-induced wilting, but visible wilting symptoms appeared much earlier in WT plants. The transgenic plants remained green after 10 days of drought stress (**Figure 4A**). Under stress conditions, the transgenic plants showed significantly reduced levels of lipid peroxidation end product, malondialdehyde (MDA) (**Figure 4B**). The MDA content was 3.5- to 2-fold lesser in transgenic plants than in WT. The groundnut transgenics expressing *MuWRKY3* maintained a higher amount of free proline and total soluble sugar content than the WT plants even after 10 days of drought stress imposition (**Figures 4C,D**), resulting in a range of 1.5- to 2.5-fold and 2.3- to 4.4-fold increase in the amount of proline and total soluble sugar contents, respectively, in transgenic lines was observed.

### Transgenic Groundnut Expressing *MuWRKY3* Increases Antioxidant Enzymes to Reduce Oxidative Stress Damage

**FIGURE 5:**
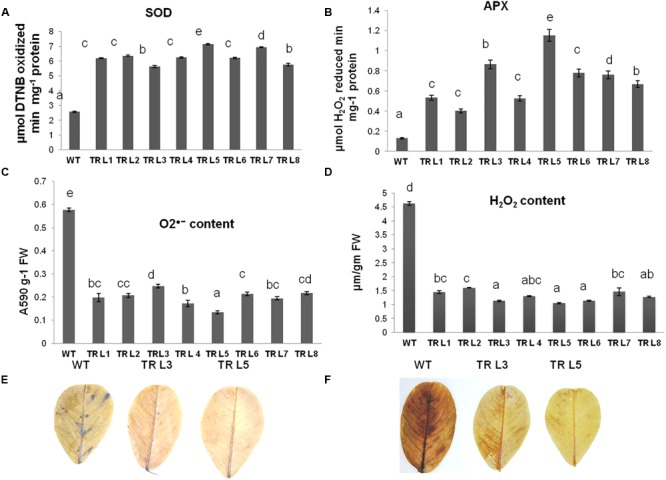
Physiological characterisation of WT and *MuWRKY3* transgenic plants under drought stress conditions. Antioxidant enzymes: **(A)** SOD **(B)** APX activity in WT and transgenic lines under drought stress conditions. **(C)** O^∙-^ anions produced in WT and transgenic plants under stress conditions. **(D)** H_2_O_2_ ions produced in WT and transgenic plants under stress. **(E)** *In situ* histochemical localization of O^∙-^ generated under drought stress using DAB. **(F)** *In situ* histochemical localization of H_2_O_2_ production under stress using NBT. Data is analyzed using SPSS version 16.0, values shown is the mean of three replicates and ±*SE* of three replicates and letters shown above the bars are significantly different at *P* < 0.05 (DMR).

The ability of transgenic groundnut plants to withstand the oxidative stress damage was studied by exposing the plants to drought stress conditions. The antioxidant enzyme activity (SOD and APX) was significantly higher in transgenic lines compared to WT. The SOD activity was three- to fivefold more in transgenic lines than in WT. Similarly, APX activity was three- to sevenfold more in transgenic plants than in WT (**Figures 5A,B**). The stress-induced production of O_2_^∙-^ and H_2_O_2_ radicals were higher in WT plants than that of transgenic lines (**Figures 5C,D**). These results were also supported by the *in situ* histochemical staining of O_2_^∙-^ with NBT and H_2_O_2_ ions with DAB. Remarkable differences were observed where WT plants accumulated higher free radicals than that of transgenic plants (**Figures 5E,F**). These results suggest that transgenic plants overexpressing *MuWRKY3* gene showed better tolerance to drought with increased antioxidant efficacy.

### Transgenic Groundnut Plants Are Stable and Showed Increased Expression of Stress-Responsive Genes

**FIGURE 6:**
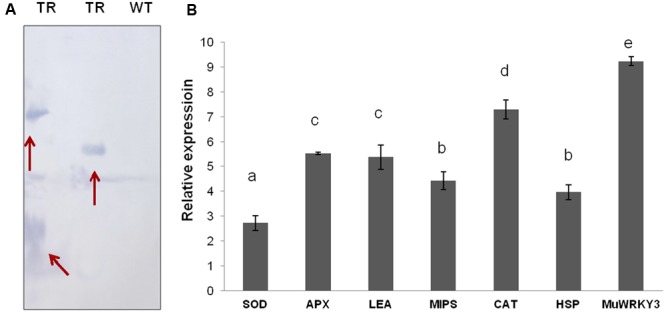
**(A)** Southern blotting analysis of transgenic plants for the detection of positive plants with stable integration (TR, transgenic line; WT, wild-type). **(B)** qRT-PCR analysis of *MuWRKY3* and stress responsive genes under drought stress conditions.

Selected groundnut transgenic plants are tested for the stable integration of gene by Southern blot analysis in a T3 generation. The variation in the integration pattern between transgenic lines demonstrated the independent transgenic events and also confirmed transgenic nature of groundnut plants (**Figure 6A**).

Further understanding of the WRKY TF role in the mechanism of drought tolerance, the selected six down stream stress-responsive gene expression was studied and compared between WT and *MuWRKY3* transgenic groundnut plants under drought stress conditions. An efficient antioxidant system is crucial to alleviate the oxidative damage caused by drought stress. QRT-PCR analysis studies used to check the gene expression of ROS-scavenging enzymes (*CAT, SOD, and APX* genes) and other stress responsive genes such as *MIPS, LEA*, and *HSP*. Drought stress increased the transcript levels of CAT, SOD, and POD in transgenic lines. The drought stress-induced expression of all six selected stress-responsive genes showed elevated expression in transgenic groundnut plants.The increases in transcript levels of 2.71-fold for SOD, 5.5-fold for APX, 7.3-fold for CAT, 4.4-fold for MIPS, 5.3-fold for LEA, 4.0-fold for HSP, 9.4-fold for *MuWRKY3* over those in WT plants (**Figure 6B**). Overall these results suggest that over-expression of *MuWRKY3* regulates the expression of downstream stress-responsive genes under drought conditions.

## Discussion

Drought is one of the major abiotic factors that accounts for 30–70 percent of crop yield loss worldwide. At whole plant level, drought stress causes a series of morphological, physiological, biochemical, and molecular changes that effect plant growth, development and productivity (Rahdari and Hoseini, 2012; Ali et al., 2014). Effect of drought on plants growth (Rampino et al., 2006), water content (Jaleel et al., 2009) decrease nutrient uptake (Selvakumar et al., 2012), decrease in photosynthesis (Rahdari et al., 2012) resulting in the severe damage and yield loss was extensively studied. Many research efforts have been made to produce the crops tolerant to drought through plant breeding and genetic engineering technologies. Transcription factors (TFs) are the ideal candidates to engineer crops for improved tolerance as they regulate many downstream functional genes (Agarwal et al., 2011). Under stress conditions the TFs interact with *cis*-elements in the promoter region of the gene and up-regulate the expression of many downstream genes imparting stress tolerance (Agarwal et al., 2011; Li et al., 2013). Overexpression of TFs imparting plant drought stress was previously reported. Expression of MYB (Seo et al., 2011; Shin et al., 2011; Nakashima et al., 2014), bZIP TF genes (Banerjee and Roychoudhury, 2017) and NAC family TFs (Shao et al., 2015) imparting drought stress tolerance was extensively studied in plants. These TFs impart stress tolerance by altering the biochemical characters like osmolyte accumulation (Wang et al., 2009; Niu et al., 2014), antioxidant enzyme production (Shi et al., 2014; Chu et al., 2016) and by the regulation of stress responsive genes (Zheng et al., 2013; Wang et al., 2015).

WRKY TF are the largest TF family proteins bind to the specific TTGAC(C/T) W-box elements in the promoters of a many plant defense-related genes (Brand et al., 2013). Based on the number of WRKY domains and the nature of their zinc-finger motif the WRKY TFs are classified into three groups. The presence of two conserved WRKY domains and a C2H2 zinc finger motifs in the WRKY3 TF gene of horsegram reveals that its a group I of WRKY superfamily proteins. Phylogenetic analysis showed that *MuWRKY3* is clustered with *GmWRKY3* from soybean. The previous studies suggest that WRKY genes play a vital role in pathogen-defense mechanisms (Dang et al., 2013; Cai et al., 2015; Sarris et al., 2015) and abiotic stress responses (Luo et al., 2013; Banerjee and Roychoudhury, 2015; Dai et al., 2015). In our study, the rapid up-regulation of *MuWRKY3* gene was induced by drought, dehydration, heat, salt, and cold stress. Similar results were reported from previous studies where multiple abiotic stresses highly induced the expression of *TaWRKY10* (Wang et al., 2013) and *TaWRKY2* genes (Niu et al., 2012).

Transcription factors are known to be located in the nucleus, where they bind to specific gene promoter sequences, thereby controlling the downstream elements. The result obtained from the subcellular localization of *MuWRKY3* showed that the YFP was located in the nucleus, suggesting that this gene function could be nuclear localized. A similar result was observed when soya bean *WRKY20* was expressed in transgenic Arabidopsis plants, where green fluorescence protein localization was observed in the nucleus (Luo et al., 2013). We employed a standardized tissue culture-independent *Agrobacterium*-mediated *in planta* transformation protocol to generate transgenic groundnut lines overexpressing *MuWRKY3* (Rohini and Rao, 2001). The selection of putative transgenic plants was carried out from the T1 generation as the primary transformants (T0) are chimeric in nature (Rohini and Rao, 2001; Kumar et al., 2009).

The previous studies has reported the improvement of drought tolerance in plants by expression of WRKYTF genes (Luo et al., 2013; Wang et al., 2013; Chu et al., 2015). An increase in the expression of the *OsWRKY30* in transgenic rice was identified in response to drought by Shen et al. (2012). The *GmWRKY20* TF gene derived from wild soybean has improved the drought tolerance in transgenic *Arabidopsis* plants through ABA-mediated signaling (Luo et al., 2013). Similarly, the overexpression of *MuWRKY3* gene in groundnut transgenics showed enhanced tolerance to drought stress. The improved cellular level tolerance achieved by this *MuWRKY3* expression and regulation of other stress-responsive genes could be partialiy attributed to delayed wilting, stay green nature, several physiological and biochemical changes in the groundnut transgenics.

Firstly, accumulation of osmolytes such as soluble sugars and free proline is an important phenomenon to facilitate the osmoregulation in response to abiotic stress (Garg et al., 2002; Singh et al., 2015). Higher accumulation of free proline and soluble sugars was observed in *MuWRKY3* transgenic plants compared to WT plants under drought stress condition. Consequently, the higher accumulation of osmolytes in the *MuWRKY3* transgenic plants may partially contribute to the enhanced drought tolerance. Similarly, the transgenic alfalfa plants overexpressing WRKY20 from *Glycine soja* have accumulated more free proline and soluble sugars under drought stress (Tang et al., 2014). *BhWRKY1* from *Boea hygrometrica* regulating the accumulation of osmolytes in response to water stress was also reported (Wang et al., 2009).

Secondly, higher accumulation of cytotoxic compounds (ROS and RCC) during drought stress can cause severe oxidative damage in plants. The lipid peroxidation induced by higher ROS accumulation produces a wide range of degradation products, such as MDA, methylglyoxal (MG), HNE, which are responsible for severe electrolyte leakage. The *MuWRKY3* transgenics showed lower levels of MDA under drought stress; this may be due to lower O^∙-^ and H_2_O_2_ levels. Abiotic stress can cause lipid peroxidation, leading to accumulation of MDA that reflect the high degree of damage triggered by stress (Sathiyaraj et al., 2011). The continuous monitoring of ROS in the cell is essential, and equilibrium between the generations of ROS and scavenging depends on antioxidative enzymes (Foyer et al., 2008; Suzuki et al., 2012). Plants with a high level of antioxidant enzymes are relatively more tolerant to abiotic stress (Peleg and Blumwald, 2011). The oxidative stress-responsive genes such as SOD, APX, and CAT catalyze the degradation of ROS produced during stress (Zheng et al., 2013; Wang et al., 2015). The *MuWRKY3* transgenic plants showed higher activity of antioxidative enzymes like CAT, SOD, and APX compared to WT plants under drought stress.). The results suggest that the over-expression of *MuWRKY3* confers drought stress tolerance in transgenic plants by reducing the effect of ROS and its accumulation.

Thirdly, to understand the specific role of *MuWRKY3* during drought stress, we studied the expression of six stress-responsive genes such as SOD, APX, CAT, MIPS, LEA, and HSP in WT and transgenic lines. The transgenic plants have shown the up-regulation of all six stress-related genes with highest transcript abundance in CAT. Wang et al. (2015) reported that the transcript levels of antioxidant genes were increased in transgenic tobacco plants expressing the *TaWRKY44* gene under drought stress treatments. Overexpression of *ThWRKY4* conferred tolerance to salt and oxidative stress by modulating ROS by expression of SOD and APX genes (Zheng et al., 2013). Up-regulation of HSP proteins like HSP (LOC_ Os0g16061) 358 and HSP (LOC_Os9g31486) was observed in rice plants expressing OsWRKY30 under drought stress (Shen et al., 2012). In the present study, we report that the expression of *MuWRKY3* in transgenic plants is up-regulating the MIPS gene under drought stress conditions. LEA proteins were known to accumulate during water stress conditions and help plants to survive (Garay-Arroyo and Covarrubias, 1999) by stabilizing macromolecules and cellular structures (Liu et al., 2013). Transgenic tobacco expressing *TaWRKY44* showed elevated expression of LEA type genes and confers drought tolerance (Wang et al., 2015). Positive correlations between expression of HSPs and heat shock (HS) tolerance have been reported previously (Burke and Chen, 2015). MIPS gene is known to involve in membrane formation, cell wall biogenesis, stress response, and signal transduction (Kaur et al., 2013; Goswami et al., 2014). Zhai et al. (2016) as reported that an myoinositol-1-phosphate synthase gene(IbMIPS1) has enhanced the salt and drought tolerance in transgenic sweet potato plants. The results indicate that HSP and MIPS genes were up-regulated under drought stress conditions in transgenic plants compared to WT plants. Up-regulation of HSP in rice plants expressing *OsWRKY30* under drought stress was previously reported (Shen et al., 2012). The gene expression studies demonstrated that *MuWRKY3* gene regulates the expression of drought stress-responsive genes. These results are supported by the previous studies that the stress tolerance achieved in transgenic plants expressing WRKY genes by up-regulation of stress-related genes (Hu et al., 2013; Wang et al., 2015).

## Conclusion

In summary, the horsegram *MuWRKY3* gene belonging to group I of the WKRY superfamily is a stress-inducible TFs. Overexpression of *MuWRKY3* in groundnut enhanced drought stress tolerance. *MuWRKY3* transgenic plants showed up-regulation of many stress-inducible functional genes involved in diverse cellular mechanisms. The oxidative stress effect is reduced due to higher expression and activity of antioxidant enzymes. The study demonstrates that *MuWRKY3* could be a candidate gene for transgenic approaches to improve the stress tolerance in crop plants for sustained growth and productivity under drought conditions.

## Ethics statement

All the experiments were approved by RCGM, Government of India No./BT/BS.17/686/2016-PID.

## Author Contributions

CS conceived and designed the experiments. KK, MP, AN, and VAR performed the research. GLR performed the transactivation assays. UL and BV produced the transgenics. AAJ performed the gene annotations. CS, GLR, and KK wrote the paper. All authors provided inputs to develop the manuscript.

## Conflict of Interest Statement

The authors declare that the research was conducted in the absence of any commercial or financial relationships that could be construed as a potential conflict of interest.

## Footnotes

1 https://web.expasy.org/protparam/

2 http://www.csbio.sjtu.edu.cn/bioinf/plant-multi/
