# Supplementary material for: A Novel WRKY Transcription Factor, MuWRKY3 (Macrotyloma uniflorum Lam. Verdc.) Enhances Drought Stress Tolerance in Transgenic Groundnut (Arachis hypogaea L.) Plants
Source: Front Plant Sci. 2018 Mar 16;9:346. doi: 10.3389/fpls.2018.00346 (PMC5864901; doi:10.3389/fpls.2018.00346)
Supplement: Supplementary file 1 [file Table_1.pdf]

Table S1: Primers used in the study for amplification, construction of vector and expression analysis (Table S1).

**gene**	**Forward primer**	**Reverse primer**
WRKY3	ATGGCTTCTGCTGCCTCTGC	TCATGGACCCATTAGTATTC
MuWRKY3 attb	AAAAAGCAGGCTTCGAAGGAGATA GAACCATGGCTTCTGCTGCCTCT GC	AGAAAGCTGGGTGTCATGGACCCAT TAGTATTC
Adapter for attb	GGGGACAAGTTTGTACAAAAAAGCA GGCT	GGGGACCACTTTGTACAAGAAAGC TGG GT
GUS	TTCGCGTCGGCATCCGCTCAGTGGCA	GCGGACGGGTATCCGGTTCGTTGG
NptII	TGA ATG AAC TGG AGG AG	AGC CAA CGT ATG TCC TGA T
SOD-RT	GAAATGGTCCAACCACTGTGACT	TGAAATGCGGTCCAGTTGAC
APX-RT	TTAGACCCATAGCCTTGCCGAAC	AGATTTCTACCAGTTGGCTGGCG
LEA-RT	ATCACTTCAAACAAAACGAGCTTTAA	TTGTGCTGTTGTCGCATATCC
MIPS-RT	CGCTCCAAGGAAATCTCCAA	TCCCCCACATATGGCACATA
HSP-RT	AAGGACATAAGTGGCAACCCTAGA	TCCCTCATACAGCGAATCAATTT
CAT-RT	TCAAGATCGGTGGAGCGAATCACA	AGGTGTTGGTAATAACATCAAGCGGGT
WRKY3-RT	ATGGGCAGAAACATGTTAAA	TGAGTAACGGCGATTTGGTT3
