# Supplementary material for: A Novel WRKY Transcription Factor, MuWRKY3 (Macrotyloma uniflorum Lam. Verdc.) Enhances Drought Stress Tolerance in Transgenic Groundnut (Arachis hypogaea L.) Plants
Source: Front Plant Sci. 2018 Mar 16;9:346. doi: 10.3389/fpls.2018.00346 (PMC5864901; doi:10.3389/fpls.2018.00346)
Supplement: Supplementary file 2 [file Image_1.pdf]

```
ORIGIN
        1 atggcttctg ctgcctctgc ttcatttcct accactgtat gcttcaatac caatacggtt
       61 gatgaccgaa aacccagctt ctttgagttt aaaccacact gtacatcaaa tatggctcct
      121 gcagaccctg acaaccatgc aagtgaaaaa tctactcaaa tagatggtca aggaaaagct
      181 caaccttttg actcgtcacc attagtaaaa aatgagatgg cagtcccttc taatgaatta
      241 agtctatcat cacctgttca aatggttaac tcaggagtta atgcccgtgt tgaaggtgat
      301 ttggatgaac tgaaccctag gagcaacata acaactgggc ttccagcatc acaagttgac
      361 aatagaggta gtggactttc cgttgcagct gagagagtat ctgctgatgg atacaactgg
      421 agaaagtatg ggcagaaaca tgttaaagga agtgaatttc cacgcagtta ctacaaatgt
      481 acacatccta actgcgaagt caagaaacta tttgaacgct ctcatgatgg gcaaatcact
      541 gagataattt acaagggaac acatgatcac ccaaaacctc aaccaaatcg ccgttactca
      601 actggaacta tcattactat gcaagaagag agatctgata aagcttcttt gactagccga
      661 gatgacagag gatccaatat atgtggccag gtgtctcatc catctgagcc tgacagtact
      721 ccagagctat cacctgcagc tacaaatgat ggtgatcaag agggtacagg atttttgtca
      781 aaccggaata gtgatgaggt tgatgatgat gatcccttct caaagcgaag aaaaatggag
      841 cttggaaatg ctgacattac tcctgtagtt aagcccatca gggagccacg ggttgttgta
      901 caaactctga gtgaggttga tatattggat gatggttacc gctggcgcaa gtatgggcag
      961 aaggtggtga gaggcaatcc taaccctagg agttattaca aatgcacaaa cacggggtgc
     1021 cccgttagaa aacatgtgga aagggcatct catgatccaa aagctgtgat aaccacatat
     1081 gaggggaaac acaatcatga tgtaccaact gcaaggaata atagttgcca tgacattgca
     1141 ggaccagtaa gtgctgctgg gggacagaca agaattaggc cggaagaaag tgataccatc
     1201 agccttgacc ttggtatggg aattagccct gctgcggaag acacaccaag cagtcaaggg
     1261 agaatgatgc tttccgaatt tggggacagc caagctcaca ccggcaattc caatttcaag
     1321 tttgtccatt cttcgacggc gccagtgtac tttggtgttc taaataacaa ctctaaccca
     1381 tatggctcta gagaaaatcc aagtgatagt acatctttaa accgttctgc ttatccctgc
     1441 ccccagaaca tgggaagaat actaatgggt ccatga
//
```

Supplementary Figure 1: Nuclotide sequence of *MuWRKY3* gene containing 1476 bp.

Compute pI/Mw

Theoretical pI/Mw (average) for the user-entered sequence:

```
        10         20         30         40         50         60
MASAASASFP TTVCFNTNTV DDRKPSFFEF KPHCTSNMAP ADPDNHASEK STQIDGQGKA

        70         80         90        100        110        120
QPFDSSPLVK NEMAVPSNEL SLSSPVQMVN SGVNARVEGD LDELNPRSNI TTGLPASQVD

       130        140        150        160        170        180
NRGSGLSVAA ERVSADGYNW RKYGQKHVKG SEFPRSYYKC THPNCEVKKL FERSHDGQIT

       190        200        210        220        230        240
EIIYKGTHDH PKPQPNRRYS TGTIITMQEE RSDKASLTSR DDRGSNICGQ VSHPSEPDST

       250        260        270        280        290        300
PELSPAATND GDQEGTGFLS NRNSDEVDDD DPFSKRRKME LGNADITPVV KPIREPRVVV

       310        320        330        340        350        360
QTLSEVDILD DGYRWRKYGQ KVVRGNPNPR SYYKCTNTGC PVRKHVERAS HDPKAVITTY

       370        380        390        400        410        420
EGKHNHDVPT ARNNSCHDIA GPVSAAGGQT RIRPEESDTI SLDLGMGISP AAEDTPSSQG

       430        440        450        460        470        480
RMMLSEFGDS QAHTGNSNFK FVHSSTAPVY FGVLNNNSNP YGSRENPSDS TSLNRSAYPC

       490
PQNMGRILMG P
```

Theoretical pI/Mw: 6.12 / 53740.10

Supplementary Figure 2: Deduced amino acid sequence of *MuWRKY3* protein showing predicted *pI* and molecular weight.

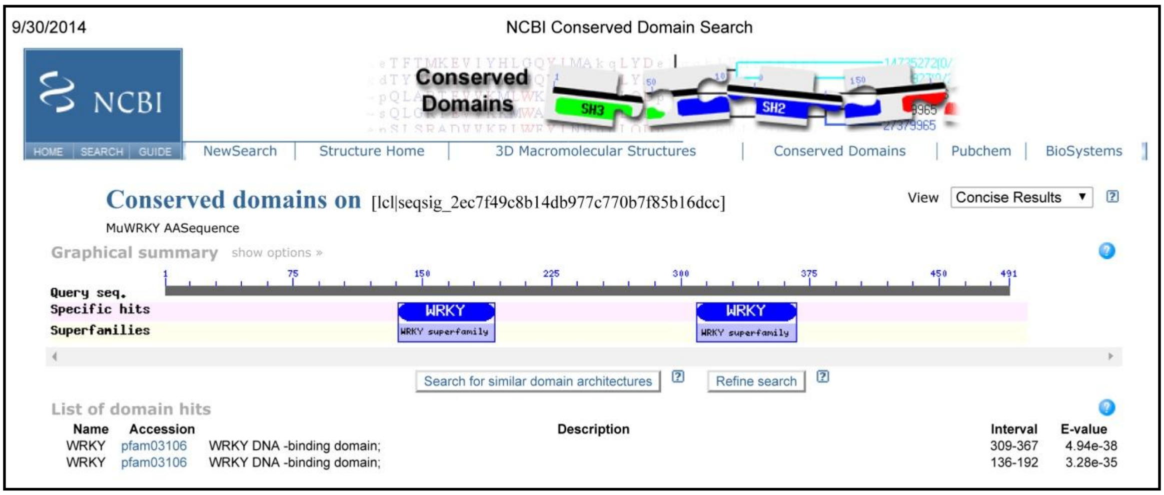

Supplementary Figure 3: Conserved domain analysis of *MuWRKY3* protein showing two domains. One towards N-terminal and another at the C-terminal region.

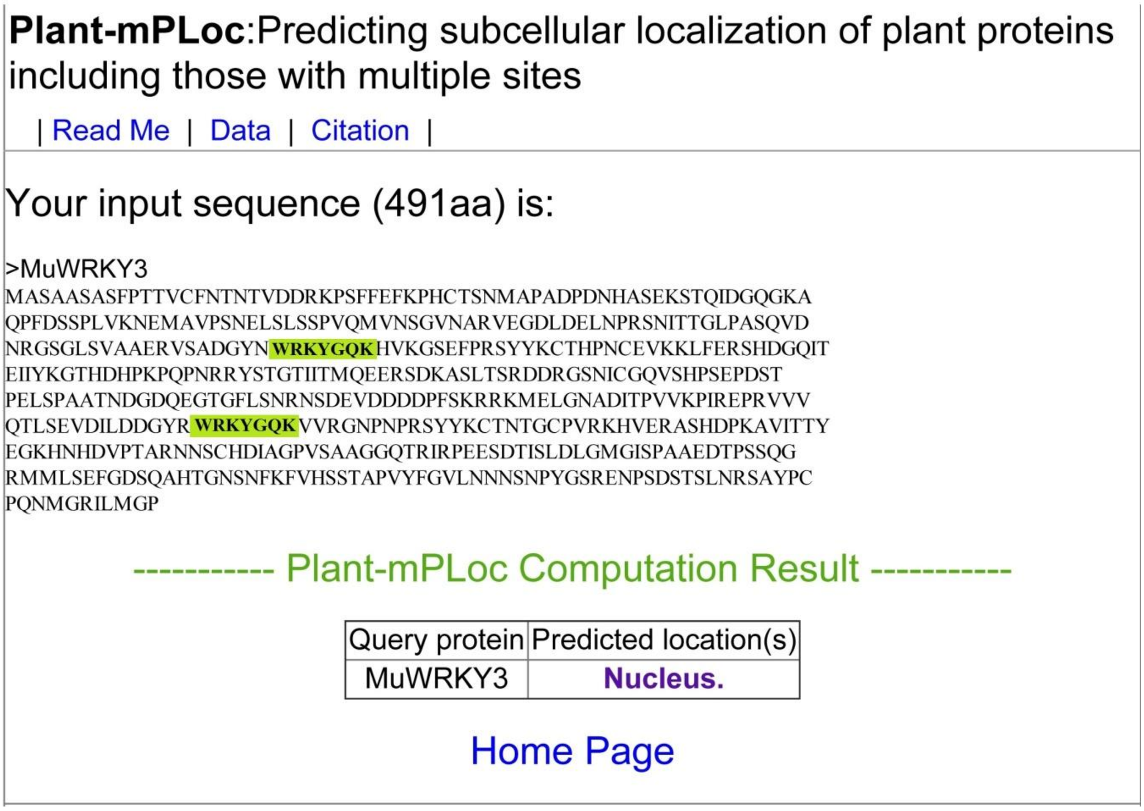

Supplementary Figure 4: Predicted sub-cellular localization of *MuWRKY3* gene showing the localization in the nucleus.

```
                   10        20        30        40        50        60        70        80
          ....|....|....|....|....|....|....|....|....|....|....|....|....|....|....|....|
NBWRKY3   --------------------------------------------------------------------------------
NTWRKY3   ----------------------------------------------------------MKKPLVHENPRKNRVIKELVDG
SMWRKY3   --------------------------------------------------------------MDNSSSDLNRAIEGLIRG
CMWRKY    ----------------------------------------------------------------METGQKWPLIKELTQG
GMWRKY3   --------------------------MASAASATFPVTTACFN-----------------TNTVDARKSSFFEFKPLNRS
MuWRKY3   --------------------------MASAASASFP-TTVCFN-----------------TNTVDDRKPSFFEFKPHCTS
NAWRKY3   ---------------------------------------------------------------------------MQSLK
ATWRKY3   MAEKEE-------KEPSKLKSSTGV-SRPTISLPPRPFGEMFFSGGVGFS---PGPMTLVSNLFSDPD-EFKSFSQLLAG
BPWRKY3   MADKEEQEVEHQQKQPSTLKSSTGAPSRPTISLPPRPFGEMFFSGGLGFS---PGPMTLVSNLFSDPD-ELKTFSQLLAG
BRWRKY3   MAENEEE----QQKQSLTSKSSTGGASRPTISLPPRPFGEMFYSGGVGFSGFSPGPMTLVSNMFSDPD-EFKSFSQLVAG
GHWRKY3   MAAEQGK------------RSKLSAPTYPTITLPPRPPIDGLFQSGSGLS---PGPMTLVSAFFSDPDSTNRSFSQLLAG
OSWRKY3   --MGGEPRARAEAAWAAALPAALVALVRDTATYTRMMHRLRMETPRGPTCQLDPCCCCCTAHVIPPPPPVSRTHATRRDA
TAWRKY3   --------------------------------------------------------------------------------
ASWRKY3   ----------------------------------------------------------------------MMTMDLMGRY
ZMWRKY3   ----------------------------------------------------------MEEVEVANRAAVESCHRVLALL
AAWRKY3   --------------------------------------------------------------------------------
VPWRKY3   --------------------------------------------------------------------------------
BOWRKY3   --------------------------------------------------------------------------------
HVWRKY3   --------------------------------------------------------------------------------
                   90       100       110       120       130       140       150       160
          ....|....|....|....|....|....|....|....|....|....|....|....|....|....|....|....|
NBWRKY3   --------------------------------------------------------------------------------
NTWRKY3   KRFAT---------------------------------QLQTLLQQPIADHGPVSADELLLKIWRSFSEAITELNTWGLA
SMWRKY3   REFTR---------------------------------RLKQIIKISGGEVENIMAEDLVAKILDSFSETLSVINNS---
CMWRKY    KEYTY---------------------------------QLKNHLDNP---MSTETCEFLVEKILNSYENAISMLNRASAI
GMWRKY3   NKVPA---------------------------------DFNNHVSKQSTQVEGPGKAQSFASSPLVESEITVPSNELSLS
MuWRKY3   NMAPA---------------------------------DPDNHASEKSTQIDGQGKAQPFDSSPLVKNEMAVPSNELSLS
NAWRKY3   NNAQS---------------------------------NGGNQYNNQSSQ------------------------------
ATWRKY3   AMASPAAAAVAAAAVVATAHHQTPVSSVGDGGGSGGDVDPRFKQSRPTGLMITQPPGMFTVPPGLSPATLLDSPSFFGLF
BPWRKY3   AMASP-AAAVAAAAVVATAH-QTPVSSVG---GSGGDVDPRFKQNRPTGLMIAQPPAMFTVPPGLSPATLLDSPSFFGLF
BRWRKY3   AMASP-----AAAAVVAGAH-QTPVSSVG--GGGDSLLDPRFKQNRPTGLMITQPPGMFTVPPGLSPATLLDSPSFFGLF
GHWRKY3   AMASP-------GAKLPYNPMDDSFMEVG--FENGGEKNSGFKQNRPLNLGVGNSP-WFTVPPGLSPSGLLNSPGLFCLS
OSWRKY3   EASVP----------------------------PPPASAAVSSRSDGTGQMAAGVTLACAAPPPLRAPRASDGGRRRGVV
TAWRKY3   --------------------------------------------------------------------------------
ASWRKY3   GRADE-----------------------------QVAIQEAAAAGLRGMEHLILQLSRTGTGTGTSES---------SLA
ZMWRKY3   SQQRD-----------------------------PALLKSVASETAEACAKFRKVAALLGSGVGHARGRFSRRVRPLGLV
AAWRKY3   --------------------------------------------------------------------------------
VPWRKY3   --------------------------------------------------------------------------------
BOWRKY3   --------------------------------------------------------------------------------
HVWRKY3   --------------------------------------------------------------------------------

                  170       180       190       200       210       220       230       240
          ....|....|....|....|....|....|....|....|....|....|....|....|....|....|....|....|
NBWRKY3   --------------------------------------------------------------------------------
NTWRKY3   FQIEEVDQADSGDRKSKDSTSELKKKKDKQGGK--------------------------------------------DRR
SMWRKY3   ----DVVVATAVEVKSPEDYSSGSCKSS-------------------------------------------------DRR
CMWRKY    HETHTFDEISPSVEVSDPEESNSPHNSHKNVFK--------------------------------------------KRK
GMWRKY3   SPVQMVSSSASAPVDVDLDEINHKGNT----------------------------------------------------A
MuWRKY3   SPVQMVNSGVNARVEGDLDELNPRSNI----------------------------------------------------T
NAWRKY3   --------------------------------------------------------------------------------
ATWRKY3   SPLQGTFGMTHQQALAQVTAQAVQGNN-VHMQQSQQSEYPSSTQQQQQQQQQASLTEIPSFSSAPRSQIRASVQETSQGQ
BPWRKY3   SPIQGAFGMTHQQALAQVTAQAVQGNG-VQIQS--QSEYPSSTQQQET-------------SSEPMSQLPA------LAQ
BRWRKY3   SPIQGSFGMTHQQALAQVTAQAVQGNS-IQMQS------ESSTQQQTL--------------LTEVPSLPAP------AQ
GHWRKY3   P--QSPFGISHQQALAQVTAQAALVQSHVHAQPEYQTLSAAGSLEPSIP---------PSSGNPEETSQQMLSSDPQSSA
OSWRKY3   KGGAGTDTCRSPQRLNVRPRERERVRACVRARAKN----------------------------------------HEHGQ
TAWRKY3   --------------------------------------------------------------------------------
ASWRKY3   GASEPAAQGQQQQQQVDCREITDMTVS--------------------------------------------------KFK
ZMWRKY3   GHKSPVGSGGNNPVEMLPSAAAVTSPS--------------------------------------------------PST
AAWRKY3   --------------------------------------------------------------------------------
VPWRKY3   --------------------------------------------------------------------------------
BOWRKY3   --------------------------------------------------------------------------------
HVWRKY3   --------------------------------------------------------------------------------
```

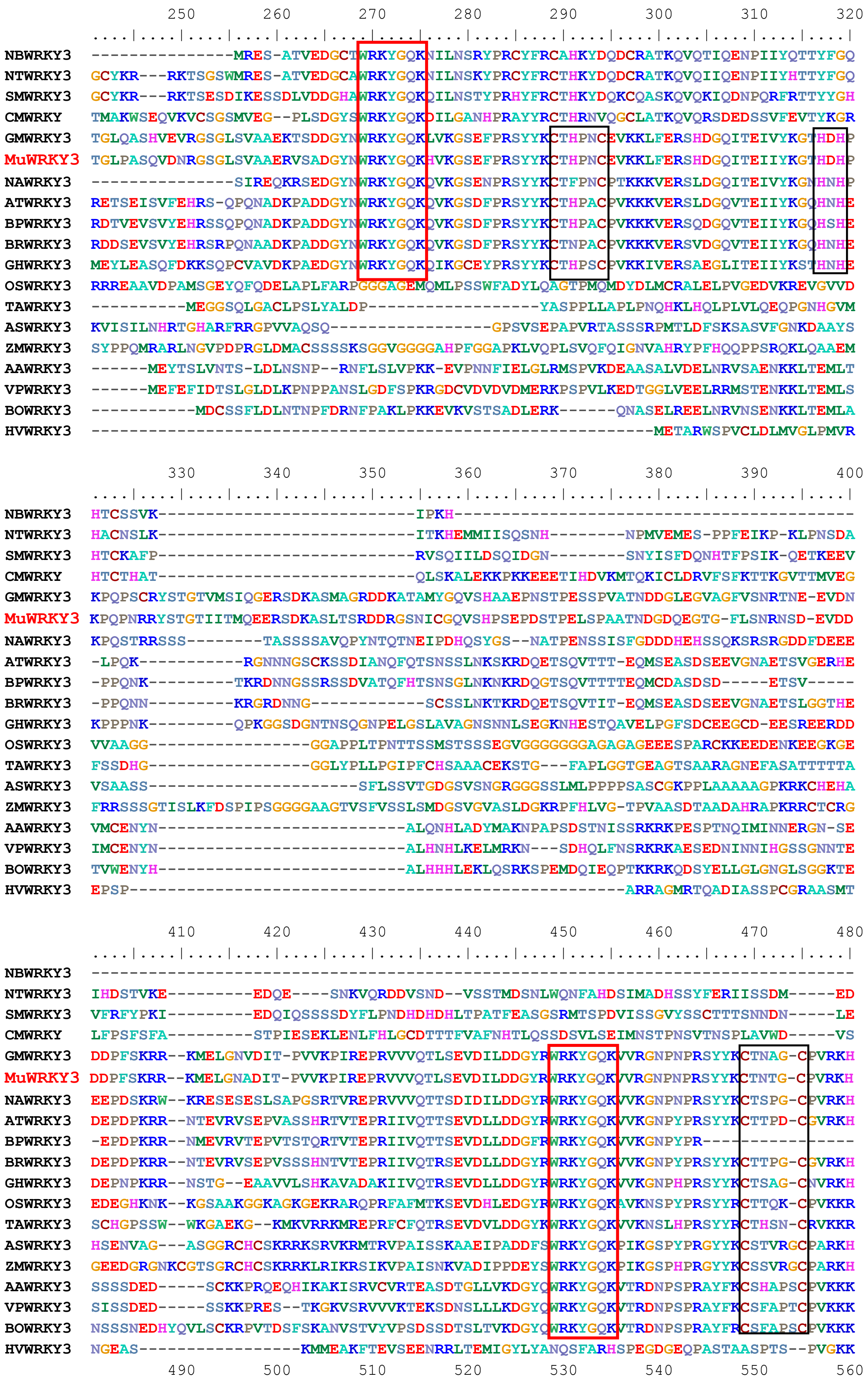
NBWRKY3
NTWRKY3
SMWRKY3
CMWRKY
GMWRKY3
MuWRKY3
NAWRKY3
ATWRKY3
BPWRKY3
BRWRKY3
GHWRKY3
OSWRKY3
TAWRKY3
ASWRKY3
ZMWRKY3
AAWRKY3
VPWRKY3
BOWRKY3
HVWRKY3

```
         ....|....|....|....|....|....|....|....|....|....|....|....|....|....|....|....|
NBWRKY3  --------------------------------------------------------------------------------
NTWRKY3  FDK-FSD--FHFDEAIEFS-------------------------------------------------------------
SMWRKY3  IDIDFEEGLWNFDQV-----------------------------------------------------------------
CMWRKY   LDHVDINGNFLFDVTDSEMFV-----------------------------------------------------------
GMWRKY3  VERASHDPKAVITTYEGKHNHDVPAARN-SSHDMAVP-AAAAGGQTRTKLEESDTISLDLGMGISSAAEHRSNGQGKMLH
MuWRKY3  VERASHDPKAVITTYEGKHNHDVPTARNNSCHDIAGP-VSAAGGQTRIRPEESDTISLDLGMGISPAAEDTPSSQGRMML
NAWRKY3  VERASQDIRSVITTYEGKHNHDVPAARGSGINRPVAPSITYNNGANAVRPFVTSQIPLQS--------------------
ATWRKY3  VERAATDPKAVVTTYEGKHNHDVPAARTSSHQLRPNNQ--------------HNTSTVNFN-------------------
BPWRKY3  --------------------------------------------------------------------------------
BRWRKY3  VERAANDPKAVITTYEGKHNHDIPASRTSSHQLRPNN----------------NLSTVNLN-------------------
GHWRKY3  VERASSDPKAVITTYEGKHNHDVPAARNSSHNTVNNSLPQPKQQHDAVAEKHSLLQEIDFR-------------------
OSWRKY3  VERSYQDPAVVITTYEGKHTHPIPATLRGSTHLLAAHAQAAAAAAAAHQLHHHGHHGHHGMAPPLPLGSGAAA------
TAWRKY3  VERLSEDCRMVITTYEGRHTHTP----------------------------------------------CSDDD------
ASWRKY3  VERDPSDPSMLIVTYEGDHRHTPGDQEAAAALTPLPELHKL---------------------------------------
ZMWRKY3  VERCVDDPAMLIVTYEGEHGHTQLPAQPSTQT------------------------------------------------
AAWRKY3  VQRSVEDQSILVATYEGEHNHPSQSKHEQASSGLNRTVT-STTLGSASLSSSGPTITLDLTTPPKPPTNPSDETKVGGDR
VPWRKY3  VQRSAEDQSLLIATYEGEHNHQ-QPSPVEVSLGFNRAATPASVLSPASMASPRPTVTLDLIQPG--LANNTAKNSVHDVK
BOWRKY3  VQRSAEDPSVLVATYEGTHNHL-------GPNGSEGDVT-------SQVGSS--TVTLDLVRSG----------------
HVWRKY3  RSRES---------------------------------------------------------------------------
                     570       580       590       600       610       620       630       640

         ....|....|....|....|....|....|....|....|....|....|....|....|....|....|....|....|
NBWRKY3  --------------------------------------------------------------------------------
NTWRKY3  --------------------------------------------------------------------------------
SMWRKY3  --------------------------------------------------------------------------------
CMWRKY   --------------------------------------------------------------------------------
GMWRKY3  SEFGDTQTHTSSSNFKFVHTTSAPVYFGVLNNSSNPFGSRDNRSDGPSLNRSSYPCPQSMGRILMGP-------------
MuWRKY3  SEFGDSQAHTGNSNFKFVHSSTAPVYFGVLNNNSNPYGSRENPSDSTSLNRSAYPCPQNMGRILMGP-------------
NAWRKY3  -----IRPHQSPFTLEMLQKPSNYNGFSGYANSEDSYENQLQDNNVFSRAKDEPRDDMFMDTLLC---------------
ATWRKY3  -------HQQPVARLRLKEEQIT---------------------------------------------------------
BPWRKY3  --------------------------------------------------------------------------------
BRWRKY3  -------QQQTVARLRLKEEQIT---------------------------------------------------------
GHWRKY3  -------NVQGPAVLRLKEEQIRV--------------------------------------------------------
OSWRKY3  ----QFGRSSGIDVLSSFLPRAAAAHHGMTTMGGAAATTTTSHGLNSAISGGGGVSSETTSAVTVAASAQPSSPAALQMQ
TAWRKY3  ----AGGDHTGSCAFTSF--------------------------------------------------------------
ASWRKY3  --------------------------------------------------------------------------------
ZMWRKY3  --------------------------------------------------------------------------------
AAWRKY3  RVDTPEFQQFLVDQMASSLTKDPSFKAALAAAISGRMVQQNQSQKW----------------------------------
VPWRKY3  --EKPVVQQFLVEQMASSLTRDPSFTAALAAAISGRILDQTQIEKW----------------------------------
BOWRKY3  ---QGTMQEVLVQQMASSLTKDSKFTAALAAAISGRLMEQSRT-------------------------------------
HVWRKY3  --------------------------------------------------------------------------------
             650       660

         ....|....|....|....|....|
NBWRKY3  -------------------------
NTWRKY3  -------------------------
SMWRKY3  -------------------------
CMWRKY   -------------------------
GMWRKY3  -------------------------
MuWRKY3  -------------------------
NAWRKY3  -------------------------
ATWRKY3  -------------------------
BPWRKY3  -------------------------
BRWRKY3  -------------------------
GHWRKY3  -------------------------
OSWRKY3  HFMAQDLGLLQDMLLPSFIHGTNQP
TAWRKY3  -------------------------
ASWRKY3  -------------------------
ZMWRKY3  -------------------------
AAWRKY3  -------------------------
VPWRKY3  -------------------------
BOWRKY3  -------------------------
HVWRKY3  -------------------------
```

Supplementary Figure 5: Multiple sequence alignment with amino acids of different WRKY3 gene with *MuWRKY3*.

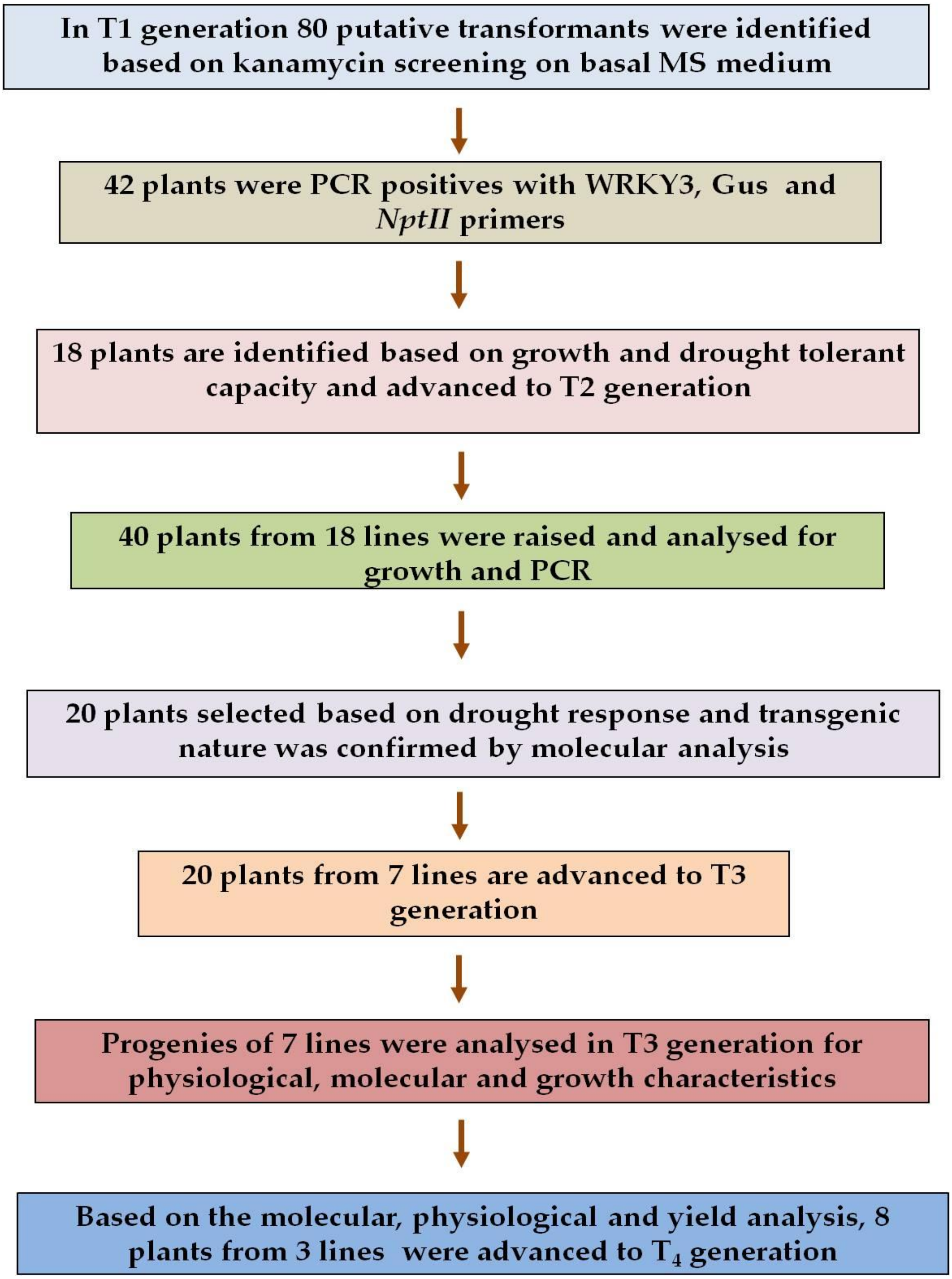

Supplementary Figure 6: Image showing the flow of events in generation of transgenic plants, analysis and advancement to next generation.
